# CIC-Rearranged Sarcomas: An Intriguing Entity That May Lead the Way to the Comprehension of More Common Cancers

**DOI:** 10.3390/cancers14215411

**Published:** 2022-11-02

**Authors:** Caterina Mancarella, Marianna Carrabotta, Lisa Toracchio, Katia Scotlandi

**Affiliations:** 1 Laboratory of Experimental Oncology, IRCCS Istituto Ortopedico Rizzoli, Via di Barbiano, 1/10, 40136 Bologna, Italy; 2 Department of Experimental, Diagnostic and Specialty Medicine (DIMES), University of Bologna, 40126 Bologna, Italy

**Keywords:** CIC-rearranged sarcoma, CIC-DUX4, Ewing-like sarcoma, DUSP6, IGF system, MAPK, ETV1/4/5

## Abstract

**Simple Summary:**

In this review, we aim to summarize the current clinical and biological knowledge for patients affected by a rare soft-tissue sarcoma, the CIC-rearranged sarcoma, to highlight novel treatment perspectives and to remark the need of innovative clinical trials. CIC-rearranged sarcoma is still entrapped in unspecific therapeutic regimens which result in poor prognosis and high incidence of recurrence. The comprehension of the biology of this tumor and the exact mechanisms of action of its molecular markers are mandatory to identify specific cancer vulnerabilities to exploit for therapy. Clinical research for rare tumors is complicated by the difficulties in patients’ recruitment and the lack of interest of Big Pharma in the development of new treatments. An intriguing perspective is given by the identification of CIC and its targets dysregulation as a common determinant of different tumor types, which might fasten the development of effective mechanism-based therapies.

**Abstract:**

Capicua transcriptional repressor (CIC)-rearranged sarcoma, belonging to the undifferentiated round cells sarcoma family, is characterized by high metastatic rate and poor chemo response. CIC sarcoma represents a new entity harboring the recurrent chromosomal translocation between *CIC* and, in most of the cases, *DUX4*. CIC-DUX4 imposes a CIC-specific transcriptional signature, which drives cell transformation, proliferation, and migration. While the discovery of the fusion represented the first evidence of a role of CIC in cancer, a complete comprehension of CIC-rearranged activity is still required before providing new potential avenues for therapy. To date, a specific and effective treatment for CIC sarcoma has yet to be defined. In this review, we initially highlight the clinical features and pathogenesis of CIC-rearranged sarcomas along with current therapeutic approaches and then focus on the specific oncogenic mechanisms driven by the CIC-rearrangement. We discuss novel therapeutic options evoked by the aberrant relations of CIC-DUX4 with the IGF system, DUSP6, P300/CBP, and CCNE1. We also discuss how different mutations involving *CIC* might converge on a common upregulation of CIC-target genes across human cancers. A deeper understanding of the oncogenic mechanisms driven by the chimera CIC-DUX4 might provide novel therapeutic opportunities with a general impact in cancer.

## 1. Introduction

Soft tissue sarcomas (STS) are malignant tumors originating in the mesenchyme and including more than 80 different subtypes, as reported by the World Health Organization (WHO) 2020 Classification of Tumors [1]. STS represents a group of complex diseases with many uncertainties regarding the biology and optimal clinical management. For patients dealing with these tumors, even receiving a correct diagnosis can be a challenge and this is particularly true for ultra-rare variants. The recent establishment of new genome-wide analysis techniques has led to the identification of new translocation-associated sarcoma types, and the recognition of distinct pathological entities. An example is given by undifferentiated round cell sarcomas [2], which show morphological similarity to Ewing sarcoma (EWS) but do not harbor EWS-specific translocations [3]. These tumors were initially named ‘Ewing-like’ sarcomas; however, they show immunohistochemical, genetic, and clinical differences from EWS. Genetically, these tumors lack the molecular hallmark of EWS, the *Ewing sarcoma breakpoint region 1- avian Erythroblastosis virus Transforming sequence* (*EWSR1-ETS*) fusion and include four distinct entities: Capicua (CIC)- and BCL6 corepressor (BCOR)-rearranged sarcomas, EWSR1-non-ETS sarcomas, and unclassified undifferentiated round cell sarcomas. The two most frequent entities are CIC-rearranged sarcomas [4,5,6,7] and BCOR-CCNB3 sarcomas [7,8]. Other even more uncommon tumors are characterized by rearrangement of *BCOR* with other partner genes [9] or by *BCOR* internal tandem duplication (*BCOR*-ITD) [10]. In addition to these entities, an additional ultra-rare subset has been defined by fusions between EWSR1 or FUS with non-ETS partners, such as EWSR1-NFATC2, FUS-NFATC2, and EWSR1-PATZ1; nevertheless, in a relevant fraction of undifferentiated round cell sarcoma, no detectable translocations have been documented so far and they are still grouped together [11]. In this review, we focus on the Capicua transcriptional repressor (CIC)-double homeobox 4 gene (DUX4) sarcoma (CDS), a high-grade sarcoma with poor outcome [4,5]. Among CIC-rearranged tumors, CIC-DUX4 fusion is the most frequent and it results from a gene fusion between *CIC* (19q13) and one of two *DUX4* retro-genes (4q35 or 10q26). The natural history and the clinical behavior of this tumor entity are still poorly defined, mainly due to the rarity of the disease. Furthermore, the optimal clinical management of CDS is also undefined both in the localized and advanced settings. However, recent molecular and genetic characterization of CDS has helped in reaching a more accurate diagnosis and highlighted novel potential molecular targets, thus expanding the range of therapeutic opportunities. The identification of rearrangements in *CIC* gene in CDS has paved the way for a better understanding of the role of *CIC* in more common cancers and questioned whether patients with CIC-rearranged sarcomas should be included in basket trials with patients who have different types of cancer, rather than with patients diagnosed with EWS or soft-tissue sarcomas.

## 2. Description of CIC-Rearranged Sarcoma

### 2.1. Epidemiology

CIC sarcoma represents a rare disease, accounting for less than 1% of all sarcomas. CDS occurs mostly in children and young adults (15–35 years), with median age in the second decade, but even elderly adults may be affected by the disease. In fact, a wide age range at diagnosis has been described, with a slight male predominance [5,6,12]. Most tumors have a predilection to occur in the soft tissue (87%), with the most frequent primary tumor location in the limb, followed by trunk, and head and neck region [4]. Occurrence in viscera, including brain, or in bone, as primary localization, is extremely rare, with 10% or 3% frequencies, respectively [4,5]. The ultra-rare variant CIC-NUTM1 seems to have distinct anatomic tropism for the axial skeleton [13]. The most common metastatic sites are the lungs, bone, liver, brain, and lymph nodes and around 40% of cases are diagnosed at metastatic stage [14].

### 2.2. Clinical Features and Treatment

Currently, patients with CIC-rearranged sarcoma are routinely treated in the same way as EWS, with a neoadjuvant and adjuvant anthracycline-based polychemotherapy regimen, surgery, and radiotherapy. Although some sporadic cases of good response to chemotherapy or radiation therapy have been reported [15,16], the prognosis of CIC sarcomas is poor. The 5-year overall survival is around 50%, which is significantly lower than the 80% 5-year overall survival of EWS patients [4,14,17]. Around 40% of CIC sarcoma patients present metastasis at diagnosis, mainly involving the lung, and patients with metastatic disease at diagnosis display an unfavorable clinical outcome compared to patients with localized disease [4,14,17]. No significant effect on survival has been observed when considering age, sex, and tumor size [4,14,17]. Available data are limited to relatively small, single-institution retrospective series but they consistently indicate that CIC sarcomas are less chemo-sensitive than EWS. This is shown by the high frequency of relapse in localized disease and short durations of treatment response in the advanced setting. In addition, histologic response to chemotherapy, evaluated according to tumor necrosis grade, indicated a minority of CIC patients (around 30%) displaying a good response to the treatment [4], which is lower than the 49% of good response in EWS [18]. Considering other non-cytotoxic therapeutic approaches, no signs of activity were identified for those few patients treated with tyrosine kinase inhibitors (regorafenib, pazopanib) or immunotherapy (pembrolizumab), who still experienced rapid disease progression [14]. Despite the limitation of these retrospective studies, the evidence from the clinic indicates that CIC-rearranged sarcoma represents a novel and discrete tumor entity, which displays a decreased histological response to standard chemotherapy compared to EWS.

### 2.3. Morphological and Genetic Features

The diagnostic accuracy is mandatory for the optimal management of CIC-rearranged sarcomas. Patients should be referred to expert sarcoma centers where pathologists can combine histology, immunohistochemistry, and molecular analysis to provide a precise diagnosis.

Morphologically, CIC-rearranged sarcoma cells feature mild-to-moderate pleomorphism. Areas with cells showing vesicular nuclei and distinctive nucleoli are present and neoplastic cells relatively often organize themselves in a lobular growth pattern, associated with presence of fibrous septa (for details see [19]). Mitotic count is generally high, in keeping with the high malignancy of these tumors. The expression of CD99, a hallmark of EWS, is often patchy with a cytoplasmic diffuse expression pattern, lacking the strong, diffuse membranous pattern observed in EWS [5]. In contrast, nuclear expression of DUX4 is consistently present [20], as well as the expression of ETS variant transcription factor (ETV) 4 and ETV5 [21,22], as a consequence of CIC-DUX4-induced transcription [23], and of Wilms tumor 1 (WT1) [24,25]. Although not entirely specific, the detection of ETVs and WT1 proteins is useful for the differential diagnosis of CIC-rearranged sarcomas. Other molecular markers that may help to distinguish CDS from EWS are cyclin D2 (CCND2) and Mucin 5AC, Oligomeric Mucus/Gel-Forming (MUC5AC) [12].

Although immunohistochemistry may help the diagnosis of CIC-rearranged tumor, the identification of molecular features is now the standard. Molecular analysis confirms the diagnosis of CDS by demonstrating the presence of specific fusion transcripts involving *CIC*. In addition to *DUX4*, *CIC*-fusion partners include *FOXO4*, *NUTM1*, and *NUTM2A* [13,26]. Precise identification of these chimeras may be relevant since, for example, CIC-NUTM1 sarcomas show distinct anatomic tropism for the axial skeleton and unfavorable behavior compared with classic CIC sarcoma [27]. In addition to the chimera, CIC-rearranged sarcomas demonstrate frequent c-*MYC* amplification and trisomy of chromosome 8, where *c-MYC* is located [28]. The impact of this alteration on the pathogenesis and/or progression of CIC-rearranged sarcomas is still unknown.

**Figure 1:**
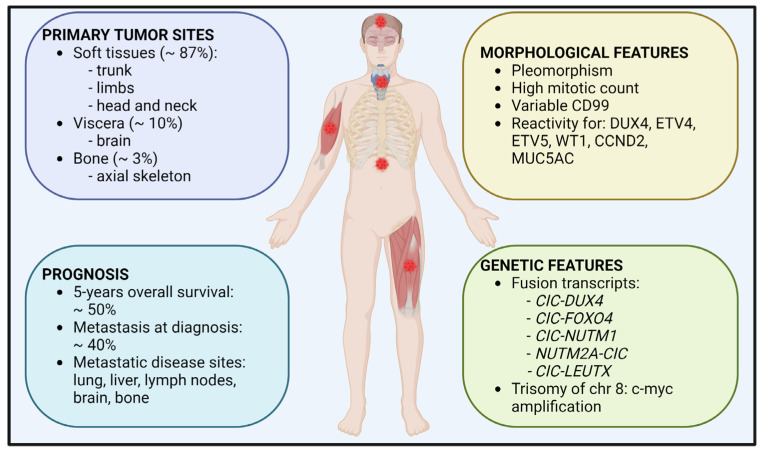
Clinical features of CIC-rearranged sarcomas. The sites of primary localization, prognosis information, along with morphological and genetic features are shown.

Major clinical characteristics of CIC-rearranged sarcomas are graphically summarized in Figure 1.

## 3. Should CIC-Rearranged Sarcomas Be Studied Together with Other CIC-Deregulated Tumors?

The discovery of the CIC-DUX4 fusion in a subset of sarcomas dates back to 2006 [23]. This was the first direct evidence that CIC contributed to cancer. Since then, the presence of cancers associated with CIC alterations has greatly expanded. In some cases, alteration of CIC defines a novel subtype-specific genetic event.

The CIC–DUX4 chimera fuses around 90% of wild type CIC, which is a transcriptional repressor [29], to the C-terminal of DUX4, a double homeobox transcription factor [23,30]. The CIC–DUX4 protein has transforming activity in NIH3T3 fibroblasts or embryonic mesenchymal cells [12,23,31]. In these models, CIC–DUX4 acts as a dominant oncogene capable to promote tumor growth and metastasis.

The DUX4 protein is composed of two N-terminal DNA-binding homeodomains, and the C-terminal domain, which activates gene transcription by recruiting the histone acetyltransferases P300/CBP [32]. While DUX4 is normally expressed during embryonic development, it is epigenetically silenced in somatic tissues but may be re-expressed in various solid cancers (for a review please refer to [30]). Functions of DUX4 in cancer are still elusive. As well as a possible role in the regulation of cell death [33,34], a role for DUX4 in immune evasion was reported [35]. Further studies are needed to clarify the impact of its expression in tumors.

*CIC* is an ortholog of the *Drosophila melanogaster Capicua* gene and it is evolutionarily conserved from *Caenorhabditis elegans* to humans [36]. *CIC* harbors two conserved domains, the high mobility group (HMG)-box and C1 domain, cooperatively involved in DNA binding [37]. Through the binding to specific DNA locations, CIC influences the transcription of its target genes. In particular, it acts as a transcriptional repressor playing a critical role in regulating several important physiological processes such as brain and lung development, T cell differentiation, abdominal wall closure during embryogenesis, and neural stem cell homeostasis (for a review see [38]). In parallel, CIC is involved in the pathogenesis of various diseases.

**Figure 2:**
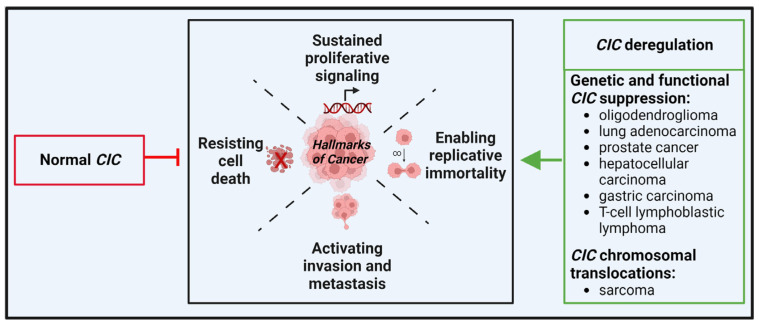
Schematic representation of CIC significance in cancer. Normal CIC acts as an oncosuppressor by inhibiting cancer onset and progression through repression of gene transcription. On the contrary, CIC deregulation promotes the reported hallmark features of cancer. Depending on the tumor type, as listed, CIC is: 1. suppressed or inactivated, following its genetic or functional alteration; or 2. transformed into a chimeric aberrant transcriptional activator by chromosomal translocation.

In cancer, wild-type CIC functions as a tumor suppressor, by repressing the transcription of specific target genes. However, genetic and functional alterations can lead to CIC loss- or gain-of-functions that ultimately sustain tumorigenesis and tumor progression in both hematological and solid cancers. A schematic representation of the role of CIC in cancer including subtypes-specific alterations is depicted in Figure 2.

In CIC-rearranged sarcomas, the chimeric proteins usually retain most part of CIC, including the C1 domain. The CIC–DUX4 fusion proteins recognize CIC binding elements in target promoters and activate instead of repress gene expression via the DUX4 activation domain [23,37]. Indeed, the chimeric proteins transcriptionally activate the expression of several CIC target genes, including: the *Polyoma virus enhancer activator* (*PEA*) *3* family genes, which encode the oncogenic transcription factors *ETV1*, *ETV4*, and *ETV5*; *Cyclin E1* (*CCNE1*) and *CCND2*; *MUC5AC* [12,31]; and are associated with sustained activation of insulin-like growth factor (IGF) signaling [39,40].

Transcriptional analysis of CIC-DUX4-bearing human tumors and experimental models clearly indicate that these sarcomas are distinct from EWS [4,12,39] and may be more similar to other CIC-driven tumors. Drastic upregulation of target genes such as *PEA3* family genes was indeed observed in other tumors due to deregulation of CIC functions. For example, inactivation of CIC in adult mice causes T-cell lymphoblastic lymphoma (T-ALL) [41,42]. Transcriptome analysis of T-ALL by RNA sequencing (RNA-seq) revealed a variety of highly derepressed CIC targets, including the transcription factors *ETV4* and, to a lesser extent, *ETV5*. *CIC* inactivating mutations were observed in brain tumors. In particular, recurrent mutations in *CIC* were found in around 50% of oligodendrogliomas, as a result of the 1p19q codeletion [43,44]. Most of these inactivating mutations cluster in the HMG-box and C1 domains that are implicated in repression of CIC targets, defining an aggressive subset of gliomas [44]. *CIC* mutations have subsequently been associated with advanced stage lung adenocarcinomas [45], metastatic progression of prostate cancer [46], gastric adenocarcinoma [45], and hepatocellular carcinoma [47]. For details, see the review by Kim et al. [48]. Taken together, this evidence indicates different mechanisms on CIC deregulation (inactivating mutations in oligodendroglioma, lung, gastric, hepatocellular carcinomas, and T-ALL; gain-of-functions mutations in sarcomas, including a small percentage of angiosarcomas that were found to carry CIC–LEUTX fusion [49]), but a common upregulation of CIC-target genes. *CIC* loss-of-function mutations cancel the CIC-mediated repression of its target genes, including *PEA3* genes, inducing cell migration and proliferation. In sarcomas, the fusion between CIC and DUX4 leads to the transformation of CIC from transcriptional repressor to transcriptional activator, with the consequent upregulation of its target genes such as *PEA3* genes. Thus, the CIC-ETV1/ETV4/ETV5 axis seems to be a common determinant of tumor formation and progression in CIC-rearranged tumors and it should be exploited for therapy.

In addition to mutations, CIC is regulated by the mitogen-activated protein kinase (MAPK) signaling. CIC activity is negatively regulated by the RTK-RAS-MAPK pathways, which cause CIC translocation from the nucleus to the cytoplasm and/or its ubiquitin-mediated proteasomal degradation [50]. The first evidence regarding this regulatory mechanism was initially discovered in *Drosophila* embryos [51,52] but it is now extended to human tumors [53,54]. Interaction between CIC and ERK is dynamic: on one side, CIC represses the transcription of the *dual-specificity phosphatase* (*DUSP*) *6* [55], a member of DUSP family phosphatases, which dephosphorylate and inactivate MAPK/ERK pathway [55,56]; on the other side, ERK induces CIC phosphorylation at different serine/threonine residues [57] and its translocation from the nucleus to cytoplasm [55], and/or CIC degradation in the nucleus by the nuclear E3 ligase PRAJA (PJA) 1 which polyubiquitylates CIC leading to its proteasomal degradation [53]. In both cases, ERK activation leads to subsequent de-repression of DUSP6. In CIC-DUX4 sarcomas, the negative feedback loop of ERK/CIC/DUSP6 turns into a positive feedback axis, where the CIC-DUX4 fusion product leads to overexpression of DUSP6 and shut down of ERK signaling, promoting CIC-DUX4 retention in the nucleus and its target genes transcription [55].

Other reported regulatory mechanisms underlying CIC activity are the following: i. the polyglutamine expansion in Ataxin-1 (ATXN1)/ATXN1L, which binds to CIC, mediates its stabilization thus promoting the binding of CIC to the promoter regions of its target genes. [58,59]; ii. the long noncoding RNA (lncRNA), AC006129, which binds to the promoter region of *CIC* and promotes DNA methylation-mediated *CIC* downregulation [60]; iii. several microRNAs which regulate *CIC* in different tumor types. In particular, *CIC* expression is inhibited by miR-1307, miR-93, miR-106b, and miR-375 [61,62,63].

Overall, all these mechanisms lead to rapid degradation/inactivation of CIC, thereby relieving repression of its downstream targets, including *PEA3* gene family members. In addition to the CIC-ETV1/ETV4/ETV5 axis, molecular studies investigating additional CIC target genes have identified *DUSP4*, *DUSP6*, *SPRED1*, *SPRY4*, *CCNE1*, *CCND1*, and IGF2BP3-IGF axis [12,23,31,39,64,65]. The effects of CIC regulation of various target genes in the different tumors that are affected by CIC dysregulation have yet to be defined to verify whether convergence on key downstream nodes may be critical across these specific cancer subtypes.

## 4. Therapeutic Options and Perspectives

CIC-rearranged sarcomas are treated in the same way as classical EWS (neoadjuvant and adjuvant polychemotherapy, surgery, and adjuvant radiation therapy). However, CIC–DUX4 sarcomas are less chemo-sensitive and more aggressive than EWS and the best therapeutic strategy is currently uncertain, in particular for recurrent/refractory tumors.

Translational research on patient-derived models and/or genetically modified cells [12,23,24,39,40,66,67] has identified several new potential therapeutic targets and opened new potential avenues for therapy. Generation and genetic characterization of novel patient-derived CIC-DUX4 sarcoma xenografts and cell lines successfully highlighted the importance of the insulin-like growth factor 1 (IGF1)/IGF1 receptor (IGF1R) pathway in this tumor. Nakai et al. showed autocrine activation of the IGF1R pathway in Kitra-SRS CIC-DUX4 cell line. Consequently, treatment with the inhibitor of the IGF1R kinase domains, linsitinib, attenuated cell growth in vitro and in vivo [40]. More recently, Carrabotta et al. highlighted an HMGA2/IGF2BP/IGF2/IGF1R/AKT/mTOR axis that characterizes CIC-DUX4 sarcomas and renders the tumors particularly sensitive to combined treatments with trabectedin and PI3K/mTOR inhibitors such as NVP-BEZ235 [39]. In this axis, HMGA2, a chromatin modifier, activates the transcription of the RNA-binding proteins insulin-like growth factor 2 mRNA binding proteins (IGF2BPs), which sustain *IGF2* and *IGF1R* mRNAs translation, with the autocrine activation of the downstream PI3K-AKT-mTOR signaling pathway. Trabectedin impairs HMGA2 activity by preventing its binding to promoters, thus inhibiting the transcription of *IGF2BPs* and decreasing IGF2/IGF1R signaling. The dual inhibition of the PI3K and mTOR pathway, using NVP-BEZ235, was required to completely dampen IGF downstream signaling mediators [39]. Thus, targeting the IGF1R/PI3K/AKT pathway may be a therapeutic strategy to fight this lethal cancer based on mechanistic evidence.

As mentioned above, CIC-DUX4 transcriptionally upregulates negative regulators of MAPK, including DUSP6 protein, to dampen ERK activity. Pharmacological inhibition of DUSP6, using (E/Z)-BCI hydrochloride (BCI), which blocks DUSP6 phosphatase domain, increased ERK activity, thus leading to CIC-DUX4 oncoprotein degradation [68]. Overall, this evidence indicates DUSP6 as a therapeutic vulnerability in CIC–DUX4 and potentially in CIC–NUTM1/FOXO4-driven tumors [13]. Interestingly, targeting negative regulators of the RAS-RAF-MEK-ERK pathway, including DUSP6, leads to cellular toxicity in lung adenocarcinoma [69], further sustaining the possibility that CIC-deregulated tumors have common determinants. Indeed, several studies implicate CIC in the sensitivity to EGFR and MAPK pathway inhibitors, suggesting that CIC may play a broader role in human cancer than originally anticipated. Absence of CIC causes resistance to MEK inhibition in T-ALL [41], lung and gastric cancer cell lines [70]. CIC was also identified as a determinant of sensitivity to blocking EGFR signaling in neural stem cells or NSCLC cell lines [65,71], suggesting that inactivation of CIC may counteract MAPK inhibition in human cancer. Thus, the levels of CIC activity should be considered as biomarkers to predict the sensitivity to MAPK inhibitors. For details on the interactions between CIC and MAPK signaling in cancer, please refer to the study conducted by Simón-Carrasco and colleagues [41].

Another therapeutic approach against CIC-DUX4 sarcoma was reported by Bosnakovski et al. [72]. The authors demonstrated that CIC-DUX4 requires P300/CBP to induce histone H3 acetylation, activate its target genes, and drive oncogenesis. The authors found that a selective and highly potent P300/CBP inhibitor, named iP300w, efficiently suppressed CIC-DUX4 transcriptional activity and reversed CIC-DUX4 induced acetylation. Overall, this evidence indicates iP300w as a promising therapeutic opportunity to fight this disease.

In addition, considering the dependence of CIC-DUX4 sarcoma on the CCNE-CDK2 cell cycle complex [31], the effects of pharmacological inhibition of CDK2, a CCNE1 binding partner, were investigated in CIC-DUX4 cells. CDK2 inhibition using dinaciclib suppressed the growth of patient-derived CIC-DUX4 cells inducing apoptotic cell death in vitro and in vivo. Overall, this evidence supports this class of inhibitors as potential anticancer agents in CIC-driven tumors. In addition, as part of a mechanism of adaptation to CCNE1 upregulation, and the subsequent DNA damage and unscheduled mitotic entry, CIC–DUX4 sarcoma cells become particularly dependent on the G2/M cell cycle checkpoint WEE1. Pharmacologic inhibition of WEE1 induces apoptosis in CIC–DUX4 cells, thus identifying WEE1 as a vulnerability targetable in this tumor type [73].

**Figure 3:**
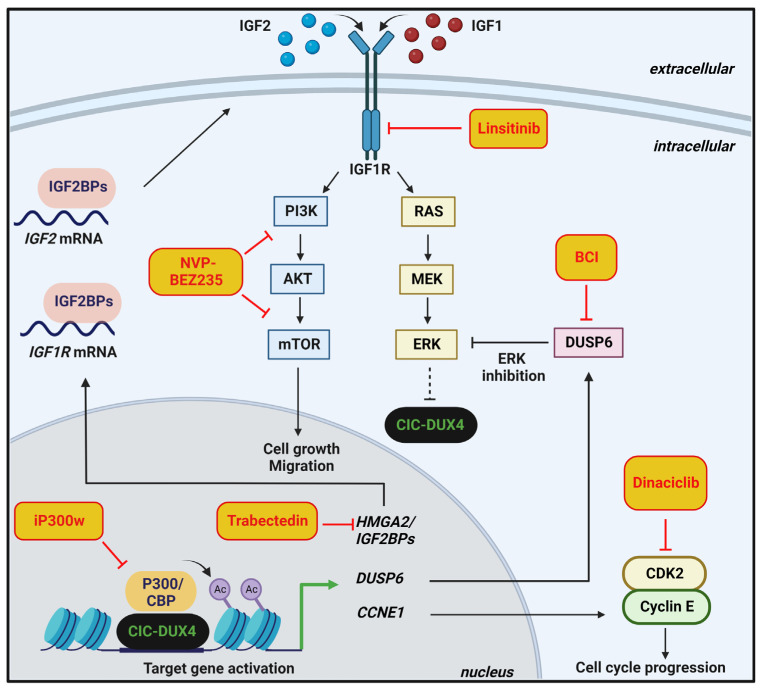
Schematic representation of CIC–DUX4-mediated oncogenic functions and related therapeutic implications in cancer. In the nucleus, CIC–DUX4 interacts with P300/CBP to induce histone H3 acetylation and transcription activation of target genes including *HMGA2/IGF2BPs* axis, *DUSP6,* and *CCNE1*. As therapeutic approaches, the P300/CBP inhibitor iP300w prevents CIC–DUX4-induced histone acetylation and reverses its functions as transcriptional activator, while trabectedin impairs HMGA2/IGF2BPs/IGF2/IGF1R axis. On the cell membrane, IGF2/IGF1 binding to IGF1R activates the PI3K-AKT-mTOR and RAS-MEK-ERK pathways. Biological responses evoked by the IGF-PI3K-AKT-mTOR axis are depicted. As therapeutic approaches, linsitinib inhibits the kinase domains of IGF1R while NVP-BEZ235 blocks both PI3K and mTOR effectors. The positive feedback loop between CIC–DUX4, DUSP6, and ERK is depicted: DUSP6 phosphatase decreases ERK activity, thus abolishing its inhibitory effects on CIC–DUX4. For treatment, inhibition of DUSP6 using BCI restores the ERK-mediated degradation of CIC–DUX4. Cyclin E binds its partner CDK2 in the cytoplasm, sustaining cell cycle progression. The use of dinaciclib, an inhibitor of CDK2, might block this oncogenic process.

In Figure 3, a graphical summary of the potential therapeutic strategies against CIC-DUX4 sarcoma is given.

## 5. Critical Issues and Perspectives

- There is no consensus on how to treat patients with CIC-rearranged sarcomas. Prospective, multi-institutional clinical studies are highly recommended as well as the creation of a prospective registry that could be useful to better describe peculiarities of these tumors and to refine the best treatment.
- Treatment of rare cancers is complicated by recruitment difficulties, and it faces a lack of interest from Big Pharma in the development of new treatments. In this respect, the possibility to design basket trials where patients from different types of CIC-deregulated tumors are commonly treated based on the presence of a specific factor may represent an interesting perspective.
- A consensus regarding the workflow for the precise diagnosis of CIC sarcomas compared to other undifferentiated round cell sarcomas is still lacking. A multimodal approach combining immunohistochemistry and molecular biology techniques, including FISH, RT-PCR, and targeted RNA-seq, needs to be employed. Tumor specimens negative for EWS-specific fusion gene should be analyzed for the panel of known fusion transcripts including CIC-rearrangement, BCOR-rearrangement, EWSR1, or FUS fusion with no-ETS partner.
- The functional impact of CIC alterations is being explored through multiple cell-based and animal model systems, leading to the potential development of novel therapies to target CIC-altered cancers. Since the challenge for investigators focused on developing new therapies in the current era is to get the right therapy to the right patient at the right time, guidelines that govern the treatment decisions and precise roadmap should be created. Operational advances in the way we conduct clinical research will help us to identify promising therapies more quickly and to move them forward toward the clinic.
- The processes mediated by the CIC–ETV1/ETV4/ETV5 axis represent the majority of our knowledge on CIC functions. Additional CIC target genes have been discovered through molecular analyses of various cancer cells; however, their role in cancer still needs to be implemented. Furthermore, future studies should explore the still unclear mechanisms by which CIC regulates the expression of its target genes.
- Most of the studies have focused on the transcriptome comparison between CIC-rearranged sarcomas and other undifferentiated round cells sarcomas, mainly EWS. A comprehensive analysis of the genomic landscape of CIC-DUX4 sarcomas, including analysis of copy number alterations (CNAs), somatic point mutations, and insertions/deletions (indels) is still lacking.
- Experimental evidence demonstrates that epigenetic modulators represent a promising therapeutic opportunity in CIC-DUX4 cellular models. However, 1. the contribution of CIC-rearrangements to DNA methylation and histone modifications, and 2. epigenetic heterogeneity among CIC-rearranged sarcomas is still unexplored and should be addressed.

## 6. Conclusions

The marriage between morphology and genetics has proven particularly useful in the definition of undifferentiated, round-cell, Ewing-like sarcomas. Particularly, genome-wide technology not only helped in achieving an accurate diagnosis but also in identifying novel potential molecular targets, expanding the range of therapeutic opportunities. Accordingly, specific inhibitors targeting some of the CIC–DUX4-related interactors have been tested in a preclinical setting and showed anti-cancer therapeutic potential. Considering the rarity of these tumors, international collaborative large studies are highly recommended. The goal is to leverage biologic insights into clinical translation as rapidly as possible to bring maximal benefit to patients who still face poor prognosis.
